# *Madhuca longifolia* as a Translational Food Biomaterial for Addressing the Triple Burden of Malnutrition

**DOI:** 10.3390/foods15152643

**Published:** 2026-07-28

**Authors:** Khushboo Yadav, Laxmi Pandey, Anuj Sharma, Mahipal Singh Sankhla, Garima Awasthi, Kumud Kant Awasthi, Theodoros Varzakas

**Affiliations:** 1 Department of Nutrition and Dietetics, University Institute of Allied Health Sciences, Chandigarh University, Mohali 140413, Punjab, India; khushbooyadav00713@gmail.com (K.Y.); lxpnduat1111@gmail.com (L.P.); 2 Department of Forensic Science, Parul Institute of Applied Sciences, Parul University, Vadodara 391760, Gujarat, India; anujs0353@gmail.com; 3 School of Basic and Applied Sciences, K.R. Mangalam University, Gurugram 122103, Haryana, India; mahi4n6@gmail.com (M.S.S.); gariimaa21@gmail.com (G.A.); 4 Department of Food Science and Technology, University of the Peloponnese, 24100 Antikalamos, Greece

**Keywords:** mahua, malnutrition, regional foods, micronutrient deficiency, food security

## Abstract

*Madhuca longifolia* plays a significant role in combating malnutrition, aligning with food security for healthier and more sustainable food options. This review highlights the latest advancements in utilizing minor forest produce and developing various mahua products, including bars, laddoos, biscuits, burfis, and pickles. This review highlights the nutritional potential of *Madhuca longifolia* in combating the triple burden of malnutrition, co-existence of undernutrition, overnutrition, and micronutrient deficiency, while focusing on the utilization of regional foods to safeguard tribal people’s livelihoods and empowerment. The regional food sources provide a viable, long-term alternative for populations with limited access to conventional food systems. Evidence regarding the health-promoting properties of *Madhuca longifolia* originates from multiple sources, including traditional ethnomedicinal knowledge, in vitro bioactivity assays, and a limited number of human investigations. A comprehensive literature search across major scientific databases identified evidence indicating that *Madhuca longifolia* is a nutritionally valuable underutilized food resource with promising applications in dietary diversification, functional food development, and sustainable nutrition systems. The reviewed evidence indicates that *Madhuca longifolia* is a nutrient-rich underutilized food resource with potential applications in dietary diversification, value-added food development, and sustainable nutrition initiatives.

## 1. Introduction

Malnutrition encompasses deficiencies, excesses, or imbalances in energy and nutrient intake that adversely affect health and well-being. Undernutrition, the most prevalent form of malnutrition, occurs when dietary energy and nutrient intake are insufficient to meet the physiological requirements necessary for growth, development, and optimal health [1,2]. Malnutrition, including undernutrition and overnutrition, remains a global health concern and is called a silent pandemic [3,4]. According to the Comprehensive National Nutrition Survey report, around 4.7 million children under 5 years of age are suffering from chronic nutritional deprivation, which is affecting their growth, survival, learning, performance in school, and productivity as adults [5]. However, as public health services, food production, and living standards have improved, the concept of malnutrition has grown less clear, with disorders such as obesity, cachexia, sarcopenia, and micronutrient imbalances becoming more widespread [2,6,7,8]. Despite reductions in the general incidence of undernutrition over the last few decades, stunting, wasting, and micronutrient deficiencies remain major public health concerns in India [9,10,11,12]. Obesity rates are higher among children and adolescents in countries with a Human Development Index score of 0.8 or above, as well as in high-income regions [13]. Obesity prevalence increased 1.5-fold from 2000 to 2011 to 2012–2023 [13]. Obese children and adolescents were more likely to experience depression and hypertension [13]. There is a rising tendency of children and adolescents to be overweight, obese, and anemic or deficient in numerous micronutrients. The phenomenon is referred to as the double burden of malnutrition [DBM] or, in some cases, the triple burden of malnutrition [TBM] within an individual [12,14,15,16]. DBM is the coexistence of undernutrition with overweight, obesity, or diet-related non-communicable diseases in an individual. TBM is defined as the existence of the following: (1) anthropometric undernutrition, (2) anemia or micronutrient deficiencies, and (3) overweight, obesity, or diet-related non-communicable diseases within a single person [12,17,18].

India is one of the world’s most populated and largest economies [12,17,18], a country with varied geographical landscapes, a plethora of cultural and ethnic customs, and an incredible range of local cuisine. The term “local” refers to the origin of food and is typically associated with the establishment of a direct relationship between the consumer and the food manufacturer [19,20]. Local food systems refer to the socioeconomic and political institutions that promote and facilitate the production, distribution, and consumption of regionally sourced foods [20,21]. Food security for the world’s population is becoming an increasingly urgent challenge. As the global population is expected to grow to 9.7 billion by 2050, food demand is estimated to increase by 70% from present levels. Traditional agriculture has serious obstacles, including limited fertile land, water limitations, climate change, and biodiversity loss. Ensuring fair access to food resources is crucial as the world’s population expands. Today, over 820 million people face hunger [22].

To enhance the translational relevance and scientific rigor of this review, a structured narrative approach was adopted to synthesize the available literature on *Madhuca longifolia*, encompassing its nutritional composition, phytochemical profile, traditional uses, biological activities, food-processing applications, and public health implications. The evidence presented has been critically evaluated according to the level of available support, distinguishing traditional knowledge from findings derived from in vitro studies, animal models, and limited human investigations. Emphasis has been placed on integrating nutritional, functional, and technological attributes within a translational framework that links plant resources to value-added food products, dietary diversification, and potential contributions toward addressing the triple burden of malnutrition. Furthermore, considerations related to processing feasibility, product scalability, regulatory requirements, compositional variability, and standardization have been incorporated to provide a balanced perspective on future utilization. The discussion has also been expanded to situate *Madhuca longifolia* within broader food-system paradigms, including sustainable diets, biodiversity conservation, One Health, and planetary health frameworks, thereby highlighting its potential role in promoting resilient, sustainable, and nutrition-sensitive food systems.

## 2. Triple Burden of Malnutrition

The coexistence of undernutrition, micronutrient deficiencies, and overnutrition in the same populations, households, and even individuals is referred to as the “triple burden of malnutrition.” Stunting, wasting, and underweight are examples of undernutrition, whereas micronutrient deficiencies, also known as “hidden hunger,” are caused by insufficient consumption of important vitamins and minerals like iron, zinc, iodine, folate, vitamin A, and vitamin B_22_. Overnutrition primarily refers to being overweight or obese. If current trends continue, 51% of the world’s population over the age of five is expected to be overweight or obese by 2035, even though one in five children under the age of five are still stunted [23].

This triple burden in the age of globalization is closely linked to the dysfunction of the world food system rather than being solely the result of personal preference. Economic globalization is linked to the coexistence of overnutrition and undernutrition, especially among the poorest households in poorer countries, reflecting unequal access to healthy diets, according to analyses of anthropometric data from 55 low- and middle-income countries. Additionally, ultra-processed foods have become more affordable and accessible than minimally processed or fresh foods, exacerbating nutrition disparities between high- and low-income groups [24].

The same article’s evidence highlights the need for systemic and policy-level solutions in addition to individual behavior modification to alleviate the triple burden. According to cohort modeling, long-term dietary adjustments that include more whole grains, nuts, and fruits and less processed meat and sugar-sweetened beverages may extend life expectancy by up to ten years. However, these changes are difficult to implement in the absence of supporting food environments. The promotion of nutrient-dense foods, taxes on sugar-sweetened beverages, biofortification of staples, and careful attention to micronutrient retention along processing chains are examples of fiscal and regulatory tools that are highlighted as crucial strategies to move food systems toward ending all forms of malnutrition in the context of accelerating globalization. Beyond conventional agricultural interventions, underutilized indigenous food resources are increasingly recognized as important components of sustainable food systems. Such species often possess nutritional, ecological, and socioeconomic advantages that can strengthen food-system resilience while diversifying dietary options. Among these resources, *Madhuca longifolia* (mahua) has attracted growing attention because of its edible flowers, seeds, leaves, and traditional importance among rural and tribal communities. Its nutritional profile and multiple value-added applications suggest potential contributions toward addressing different dimensions of malnutrition.

## 3. Potential of *Madhuca longifolia*

*Madhuca longifolia*, often known as mahua, is a deciduous tree native to the Indian subcontinent and found in tropical and subtropical regions. The tree matures around 8 to 15 years of age and can produce fruit for up to 60 years. The flowers are commonly used to create medicinal tonics that treat coughs, colds, and fevers. Mahua contains bioactive components such as flavonoids, saponins, and terpenoids that support its pharmacological properties. Research on mahua’s ethnomedicinal usage supports traditional knowledge, highlighting its potential as an antibacterial, antioxidant, and anti-inflammatory agent [25].

### 3.1. Literature Search Strategy and Review Methodology

This review was conducted using a structured narrative literature search approach. Relevant publications on *Madhuca longifolia* were identified through searches of major scientific databases, including Scopus, Web of Science, PubMed, Google Scholar, and ScienceDirect, using combinations of keywords such as “*Madhuca longifolia*,” “mahua,” “nutritional composition,” “phytochemicals,” “functional foods,” “food processing,” “health benefits,” and “food security.” Peer-reviewed articles, reviews, book chapters, and relevant reports published in English up to 2025 were considered. The retrieved literature was screened for relevance and synthesized to provide an integrated assessment of the nutritional, functional, technological, and translational potential of *Madhuca longifolia*. As a narrative review, the objective was to critically summarize current knowledge, identify research gaps, and highlight opportunities for future application and investigation.

### 3.2. Physical Characteristics

India has long been known for its diverse plant species, which have been used for medicinal and aromatic purposes. Traditional medicine is widely used for disease treatment in many nations due to its safety, cost-efficiency, and effectiveness [26,27,28]. Mahua, *M. longifolia* [Koenig] (also known as *M. indica* Gmelin; family: Sapotaceae), is a large, shady, deciduous tree that grows both wild and cultivated in central India. The tree’s blossoms, fruits, seeds, and lumber have economic value due to their application in many industries [17,28,29]. *Madhuca longifolia* flowers are small, fleshy, pale white, 2 cm long, pointy, sweet-scented, and meaty. The fruits are ovoid, fleshy, and green when immature. When fully ripe, they turn a greenish yellow. Each fruit contains three or four seeds. Mahua fruit seeds vary in length from 2.0 cm to 6.0 cm and in width from 1.3 to 1.6 cm. They are mostly ellipsoidal and the 2 cm long, brown, elongated seeds have a shiny appearance [28,29].

### 3.3. Geographic Distribution

*Madhuca longifolia*, a shady and deciduous tree is distributed in India, Nepal, and Sri Lanka [30]. Kumari et al. [31] and Purwar et al. [29] reported that this tree thrives in arid areas and can be found in tropical mixed deciduous forests across India, including West Bengal, Chhattisgarh, Jharkhand, Uttar Pradesh, Bihar, Maharashtra, Madhya Pradesh, Kerala, Gujarat, and Odisha. *Madhuca longifolia* is adaptable to a wide range of soils, but it grows best in sandy soil conditions. It can also grow in shallow, boulder-rich, clay, and calcareous soils. This species lives at altitudes of up to 1200 m, with average annual maximum temperatures of 28–50 °C and minimum temperatures of 2–12 °C. The yearly rainfall in its habitat ranges between 550 and 1500 mm [25,31]. It goes by several names, including mahua and mowarh (North). Economically disadvantaged rural and tribal communities rely heavily on this tree, which is valued for its blossoms and tora seeds [32,33,34]. Experts predict an annual yield of 45,000 M tons of mahua flowers, with each tree weighing an average of 80–320 kg. The exploration of edible flowers as a sustainable and nutritious food source reveals significant potential for enhancing global food security and nutrition [35,36].

### 3.4. Adaptability and Climatic Requirements

This plant is drought-resistant, requires plenty of sunlight, and can be suppressed in shaded areas [37]. Flowers are associated at the ends of branches from March to April. In places with an average rainfall of 1000 mm and thick loamy soils, it can attain a height of 8–10 m and a girth of 40–45 cm after roughly 10 years of artificial growth. Becoming a fully grown tree yields around 1 m^3^ of timber and 2–3 tons of fuel wood. The fruit is ovoid, meaty, greenish, 3–5 cm long, and contains 1–4 seeds. It ripens from June to August [38]. The impact of temperature on ethanol production has been thoroughly studied with temperatures typically ranging from 25 °C to 50 °C. The findings show that temperature has a considerable influence on fermentation efficiency, with departures from the ideal range potentially resulting in lower ethanol yield [39].

Traditional medicinal applications of wildflowers exist in Ayurveda and folk medicine. The edible flowers have strong medicinal effects such as antidiabetic, anticancer, anti-anxiety, anti-inflammatory, antibacterial, hepatoprotective, and neuroprotective [40]. Wild edible flowers hold unrealized potential for usage in human diets such as food, vitamins, or additives. These flowers include substances such as carotenoids, flavonoids, nutrients, and minerals, all of which contribute to their health benefits [41]. *Madhuca longifolia* possesses pharmaceutical, ethnomedicinal, and ethnopharmacological properties [42,43,44]. The use of edible flowers in functional foods is growing globally, offering new opportunities for the food and pharmaceutical industries. As a result, edible flowers have opened new options for malnutrition reduction, agricultural diversification, additional sources of income, and the protection of wild edible flower species threatened by human activity [40]. Mahua fruits, flowers, and leaves have all been used as food. In India and other Southern Asian countries, they are commonly used in vegetable dishes. Due to their natural sugar content (sucrose, glucose, fructose, arabinose, and a small amount of maltose and rhamnose), mahua flowers are used as a sweetening ingredient in traditional dishes like halva, methipuri, kheer, and burfi. Tribal people also make cakes using mahua flowers combined with grains such as rice, ragi, and jowar or root crops such as sweet potato [45,46].

## 4. Role of Mahua Flower in Combating Triple Burden of Malnutrition [TBM]

**Figure 1:**
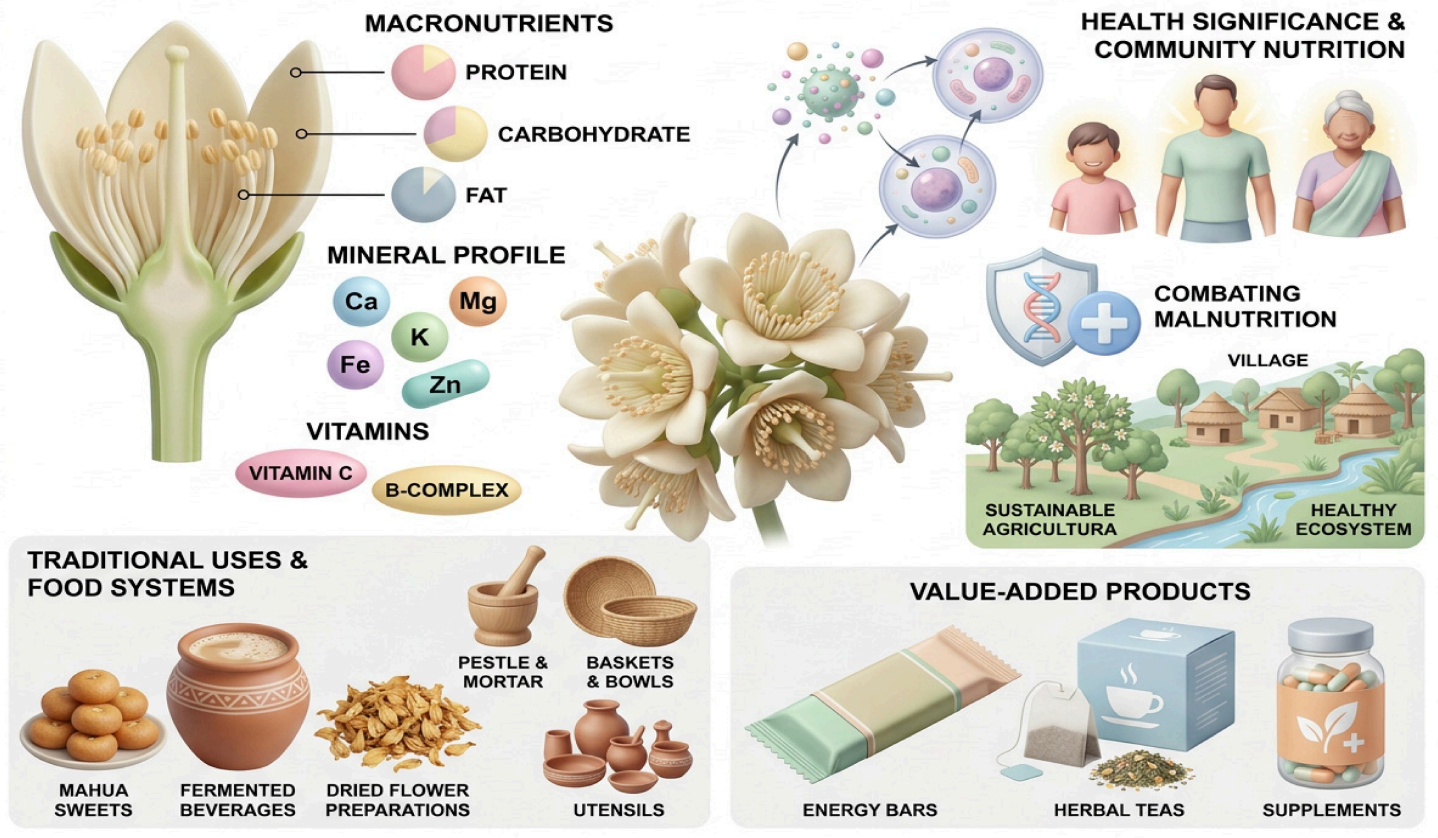
The significance of the mahua flower in combating protein-energy malnutrition. The figure summarizes the major nutritional attributes of *Madhuca longifolia* flowers, including macronutrients (carbohydrates, proteins, and fats), essential minerals (calcium, magnesium, iron, potassium, and zinc), and vitamins (vitamin C and B-complex vitamins). Traditional utilization of mahua flowers in local food systems is illustrated through the preparation of sweets, fermented beverages, dried flower products, and household items. The figure also highlights the potential role of mahua-derived bioactive compounds in supporting nutrition and health, although much of the evidence is currently derived from phytochemical, in vitro, and animal studies. Additionally, opportunities for value addition through the development of energy bars, herbal teas, and dietary supplements are presented, emphasizing the potential of mahua as an underutilized resource for dietary diversification, livelihood generation, and sustainable food systems. Figure was prepared using FigureLabs and then edited using MS-Office powerpoint.

Micronutrient malnutrition [MNM] is prevalent in developed countries but more severe in emerging economies. Developing countries, such as India, face a unique ‘double burden’ problem, with obesity in children and adolescents on one end and malnutrition and underweight on the other [47]. It affects all age groups, with small children and women of reproductive age being the most vulnerable. Indian communities of tribes continue to be the most nutritionally impoverished social groups in the country, and it is undeniable that their starvation is influenced by a variety of factors, including poverty and hunger caused by the loss of forest land and sources of income, poor rehabilitative actions, poor quality of essential food and nutrition services during critical periods of life, and geographical remoteness. According to recent survey reports, more than half of tribal children beneath the age of five in India are stunted and wasted, failing to meet their potential for growth and development. This problem is especially severe among Chenchu tribal children and may pose the greatest threat to children’s growth and development [48]. Mahua flowers contain carotene, a precursor to vitamin A. Flowers also contains plenty of elements like calcium and phosphorus. Mahua flowers also contain small amounts of proteins and lipids [35]. The tribal cultures enjoy sweet flowers during the flowering season as a substitute for expensive food grains. Off-season, sun-dried flowers are kept for future use. Fermenting flowers and adding jaggery results in an alcoholic drink used in religious ceremonies and cultural heritage. Dried flowers are used to make cakes, syrups, jams, and bread. Pancakes are a popular seasonal food among woodland dwellers. Flowers are high in sugar (54.1%), protein (6.4%), fat (0.5%), calcium (8.0%), and phosphorus (2.0%) [49]. The micronutrient levels in wild edible plants are much higher. The primary function of phosphorus [P] is the creation of bones and teeth. It influences how the body consumes carbohydrates and lipids. It also helps the body produce protein for both tissue and cell growth, maintenance, and regeneration. The RDA for phosphorus is 1250 mg for adults. In the present study, P levels ranged from 218 to 21.4 mg/100 g in *Madhuca longifolia* and Nymphaea nouchali. The human body needs calcium [Ca] for bone health and other essential functions. Calcium is also utilized to help blood arteries flow blood through the body and to release hormones and enzymes that influence practically every process within the human body. In this study, Ca values ranged from 756 to 25.3 mg/100 g in *Madhuca longifolia* and *Bambusa arundinacea*. The RDA for calcium is 1200 mg per day. *Madhuca longifolia* has the greatest value of vitamin B3 [niacin] at 4.2 mg/100 g. The body requires small amounts of water-soluble vitamins on a regular basis. These vitamins are less prone than fat-soluble vitamins to produce hazardous quantities. However, niacin, vitamin B6, folate, choline, and vitamin C have maximum dietary restrictions. Long-term exposure to high amounts of vitamin B6 has been linked to irreparable nerve damage. Vitamins were extracted from the indigenous foods consumed by the Chenchu tribes, and the vitamin ranges were found to be slightly greater than those of frequently consumed foods. The ignored and underutilized food resources found in Indigenous food settings serve as the foundation for variety in traditional and Indigenous food systems in developing countries, and they are critical for dealing with Indigenous group-specific concerns. This exists in the context of a wealth of knowledge about traditional meals that have the potential to increase micronutrient intake. The community is aware of the edible nature of a wide range of local flora and fauna. As a result, there is a need to raise knowledge about the nutritional value of these indigenous foods while efficiently packaging the message by promoting indigenous foods through nutrition education and activism. The contribution of mahua in combating malnutrition is shown in Figure 1, in which the availability of macro- and micronutrients is mentioned [48]. The potential contribution of Mahua toward addressing the triple burden of malnutrition can be conceptualized through three complementary pathways. First, its carbohydrate-rich flowers may contribute to dietary energy intake in food-insecure populations. Second, its mineral and phytochemical composition may support dietary diversification and help alleviate selected micronutrient deficiencies. Third, bioactive compounds with antioxidant and anti-inflammatory properties may support the development of functional foods aimed at reducing risk factors associated with obesity and metabolic disorders. However, the magnitude of these benefits remains dependent on consumption patterns, product formulation, bioavailability, safety, and confirmation through human intervention studies.

## 5. Potential Integration of Mahua Flower, Seed, Bark, and Leaf

### 5.1. Flower

Mahua flowers have the potential to treat eye diseases. Flowers are beneficial as an analgesic, diuretic, aphrodisiac, decadent, astringent, and cold remedy. Mahua flower juice is an effective treatment for skin diseases [50,51]. Mahua flower soaked at night on an empty stomach after a tooth in the morning will help decrease body fat, improve facial appearance, and improve intestinal bowels. It increases digestive enzymes and has properties to quickly eliminate sugar from the body. Eating a pickle prepared from its blossom improves vision, stimulates blood flow, and promotes rapid wound healing [51]. Mahua flowers are little, measuring around 2 cm in length. The color of the blossom ranges from pale white to drab, and its texture appears mushy. Mahua’s corolla appears fleshy, tubular, and pale yellow. Its distinct perfume makes it useful as a flavoring component in both food and pickle production [52,53].

Mahua flowers have a balanced profile of essential amino acids with obvious nutritional significance, as shown in Table 1. The most prevalent amino acid is leucine, which is followed by isoleucine and valine. This suggests that branched chain amino acids, which support muscle metabolism and energy balance, are strongly present. For a plant source, the levels of lysine and threonine are noteworthy, indicating potential to supplement diets based on cereals, which are frequently deficient in lysine. Methionine and cystine, two amino acids that contain sulfur, are present in moderate amounts and support antioxidant defense and protein quality. The use of mahua flowers as a useful plant-based protein ingredient to fill protein and amino acid gaps in populations at risk of undernutrition is generally supported by this amino acid composition.

### 5.2. Mahua Bark

This decoction is used to treat mucus cough, diabetes, arthritis, and bleeding gums. It also has an anti-diabetic effect [51,54]. The bark is used to cure itching, swelling, bone joining [fractures], and snake bite toxicity [55]. Bark is recommended for treating congestion and arthritis also. Bark flakes are softly heated and glued to joints [51,56]. Khare et al. [46] mentioned that to treat rheumatism, bark needs to be boiled in water, and the decoction consumed. To treat spongy and bleeding gums, gargle with 4 mL of bark extract and 300 mL of water. This mixture is also used to treat tonsillitis (both chronic and acute) and pharyngitis.

### 5.3. Seed

Fat derived from mahua seeds has numerous therapeutic applications. The seeds’ fat contains emulsifying properties and is used for treating skin diseases, rheumatism, headaches, laxatives, and piles, and as a galactagogue. It can be used as a laxative for constipation and piles, a gummy juice for rheumatism and skin problems, and an oil to treat skin diseases [41]. Detoxified mahua seed flour is a promising protein source for food and feed products [57]. Mahua seeds can be used to make defatted flour, which has high potential in baking items. The saponin produced during extraction has industrial and commercial uses. Extracted oil cake can be utilized as manure and has insecticidal effects. It produces high-quality timber wood for diverse applications. Mahua is a valuable tree for tribal and low-income communities in India because of its economic potential [58].

### 5.4. Leaves

*Madhuca longifolia* leaves have expectorant properties and can be used to treat chronic bronchitis, Cushing’s disease, verminosis, gastropathy, consumption, bronchitis, dermatopathy, rheumatism, cephalgia, and hemorrhoids [51,54]. Mahua leaf is used as medicine by crushing fresh and young leaves and applying juice to wounds. Regular application of extracts results in wound healing, highlighting mahua’s antimicrobial properties [51,59]. To heal scalds and burns, ash is mixed from leaves with ghee. Applying bark paste to the affected area relieves itching. *Madhuca longifolia* leaf extracts (acetone and ethanol) showed cytotoxic efficacy against Ehrlich ascites carcinoma cell lines by application of in vitro experiments at 200 g/mL. Both extracts were cytotoxic, although the ethanol extract showed more activity. Madhuca leaves are excellent for treating eczema, a condition caused by inflammation of the skin. To treat eczema, roast sesame oil-coated leaves were spread over a fire and applied to affected areas [46].

Table 2 shows that *Madhuca longifolia* is a multifunctional plant with clear relevance to food, nutraceutical, and biomaterial research. Different plant parts provide complementary bioactivities, ranging from antioxidant and antidiabetic effects in flowers to anti-inflammatory and wound healing actions in seed oil, bark, and leaves. The overlap between traditional uses and experimental evidence strengthens confidence in its efficacy and safety. Importantly, the whole tree offers a broad spectrum of phenolics, flavonoids, saponins, and minerals, supporting its use as a sustainable source of functional food biomaterials rather than a single compound remedy. Together, these findings position *Madhuca longifolia* as a translational bioresource that can be standardized and integrated into modern food systems to address micronutrient deficiencies and cardiometabolic disorders.

## 6. Nutritional Composition of Mahua

*Madhuca longifolia* contributes to dietary energy intake due to its high content of naturally occurring sugars, carbohydrates, and seed lipids, making it a potentially valuable food resource in regions vulnerable to food insecurity. In addition, mahua flowers and other plant parts contain vitamins, amino acids, minerals, and bioactive phytochemicals that may support micronutrient intake and dietary diversification. The presence of polyphenols, flavonoids, triterpenoids, and antioxidant compounds has also generated interest in mahua as a source of functional food ingredients. Consequently, mahua has been explored for the development of value-added products such as beverages, bakery products, confectioneries, and nutraceutical formulations, although further studies on bioavailability, safety, and clinical efficacy are required to substantiate health-related claims.

According to non-timber forest products (NTFPs), the mahua tree is a nutritional powerhouse. The mahua tree can be used for medical purposes, liquor manufacturing, biodiesel production, and food goods. mahua fat is a natural source of hard fat that can replace cocoa butter or ghee with chocolate manufacture. Mahua seed fat has emulsifying qualities and is utilized in the production of laundry soaps and lubricants [45,46,47,48,49]. Mahua flower is a rich source of nutrients such as reducing and non-reducing sugars, polysaccharides, dietary fibers, fats, proteins, vitamins, minerals, enzymes, and various organic acids. The sugar content of the dried mahua flower ranges from 40 to 70%. The sugar concentration of flowers varies by geography, being gathered, and type of Mahua [63]. The nutritional value of mahua flower is shown in Table 2. The phenolic components found and measured in mahua flower and fruit extracts included ascorbic acid, gallic acid (GA), quercetin, and myrcetin [50].

**Figure 2:**
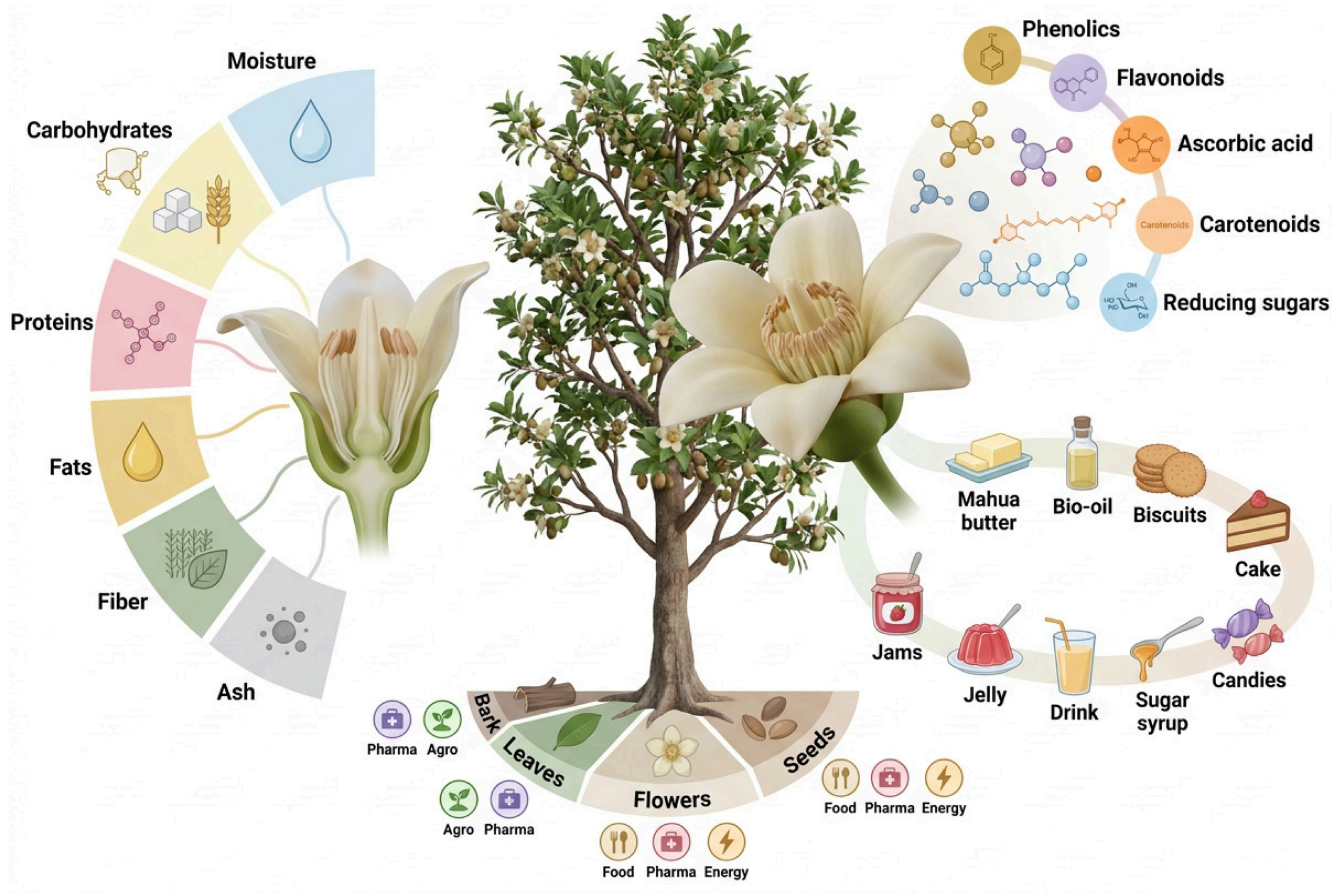
Nutritional composition, bioactive constituents, plant-part utilization, and value-added product applications of *Madhuca longifolia* (mahua). The figure provides a comprehensive overview of the nutritional and industrial significance of *Madhuca longifolia*. The left panel highlights the major nutritional components of mahua flowers, including carbohydrates, proteins, fats, dietary fiber, ash, and moisture, demonstrating their potential as a nutrient-rich food resource. The upper right section illustrates key bioactive compounds reported in mahua, such as phenolic compounds, flavonoids, ascorbic acid, carotenoids, and reducing sugars, which are associated with antioxidant and other biological activities. The lower section depicts the diverse utilization of different plant parts, including bark, leaves, flowers, and seeds, across agricultural, pharmaceutical, food, and energy sectors. The right panel showcases representative value-added products developed from mahua, including Mahua butter, seed-derived bio-oil, biscuits, cakes, jams, jellies, beverages, sugar syrup, and confectionery products. Collectively, the figure demonstrates the multifunctional role of *M. longifolia* as a source of nutrients, bioactive compounds, industrial raw materials, and value-added food products, highlighting its potential contribution to food security, sustainable utilization, and rural bioeconomy development. Created in BioRender. Antoniadou Maria. (2026). https://BioRender.com/kqxdz9q (accessed on 25 May 2026).

Mahua flowers are a nutrient-dense, high-energy food source and that is relevant for addressing undernutrition, as Table 3 demonstrates. Their caloric contribution is explained by their high carbohydrate and total sugar content, and their moderate protein content meets basic dietary protein requirements. Significant levels of calcium and phosphorus suggest a role for mineral supplementation and bone health, particularly in diets based on cereals. The vitamin profile, which includes thiamine, riboflavin, and niacin, supports energy metabolism and micronutrient adequacy despite the low fiber content. Overall, this composition emphasizes mahua flowers as a useful natural component for enriching traditional and prepared foods with vitamins, minerals, and energy. A schematic overview of nutritional composition, bioactive compounds, and bio-valorization pathways of *Madhuca longifolia* is shown in Figure 2.

### 6.1. Carbohydrate Content of Mahua

Mahua flowers were discovered to be a great source of carbohydrates, and when dried, significant changes in moisture and fat content were observed [65]. Dietary carbohydrates are chemically defined compounds that have various physical and physiological qualities, as well as positive health impacts [63]. Carbohydrates that are slowly digested in the small intestine or pass through undigested are often regarded as “high quality”. Dietary fiber is an essential indicator of carbohydrate quality [5,66]. The water-soluble polysaccharide from mahua flowers was divided into two pure parts after being filtered through Sephadex G-150. The first part included D-galactose, L-arabinose, L-rhamnose, D-xylose, and D-glucuronic acid. The anomeric structures of the various sugar residues in the polysaccharide were determined by Lungade et al. [67]. The modifiable risk factors for obesity include poor diets, physical inactivity, and hazardous alcohol consumption. The quality of carbohydrates in the diet has been intensively studied as a potential moderator of non-communicable disease and obesity risks. Carbohydrates are present in a wide variety of plant-based meals and are the primary source of energy which serves as metabolic “fuel” for the brain and other organs in the body. Monosaccharides are the building blocks of carbohydrates, which can be classified as disaccharides, oligosaccharides, and polysaccharides such as starch, based on their degree of polymerization [66,68]. Hydrolyzed and unhydrolyzed extraction were utilized to extract various polysaccharides from the mahua flower. Sucrose, rhamnose, arabinose, fructose, maltose, and glucose were identified in unhydrolyzed extraction and analyzed using paper chromatography. Galacturonic acid, together with glucose, arabinose, rhamnose, fructose, and maltose were discovered in the hydrolyzed extract [63,69].

### 6.2. Protein Content of Mahua

Proximate analysis showed that the mahua flower powder is a good source of protein. Therefore, the powder was subjected to protein analysis. Two distinct treatments were applied: TCA–acetone and alkaline treatment. After comparing the approaches, it was discovered that the TCA–acetone sample had higher protein content and greater protein extraction yield than the alkaline technique, and it also exhibited increased foaming capability. Various research has looked at the commercial potential of mahua, recognizing its wide range of applications across industries [70]. According to the recommended dietary allowances (RDAs), protein intake is 0.83 g protein/kg/day for healthy women and men [71]. Mahua provides 6.67 g of protein per 100 g, which is helpful for the fulfillment of the daily protein requirement [53,69,72,73,74].

### 6.3. Micronutrient Content of Mahua

The nutritional value of mahua includes high levels of calcium, phosphorus, and vitamin A. Breastfeeding women report that mahua enhances milk production and secretion [29]. Sugar assessment of mahua flowers and fruits revealed the presence of mannitol, dextrose, sorbitol, inositol, and raffinose. All samples had significant amounts of Ca, Mg, Na, and K [43]. Calcium (139 mg/100 g), phosphorus (137 mg/100 g), and niacin (4.80 mg/100 g) were found [63]. Mahua flowers contain vitamin C, which is responsible for the antioxidant activity [29,75]. The preliminary phytochemical screening of *Madhuca indica* extracts revealed notable variations in the distribution of bioactive constituents among different solvent systems. Ethanol and aqueous extracts showed the richest phytochemical profiles, containing terpenoids, carbohydrates, flavonoids, tannins, saponins, phenolic compounds, fixed oils, and fats. Alkaloids and glycosides were not detected in any of the extracts. The chloroform extract exhibited a comparatively limited phytochemical composition, with the presence of phenolic compounds and fixed oils only. These findings suggest that polar solvents, particularly ethanol and water, are more effective in extracting a broad range of bioactive compounds from *Madhuca indica*, which may contribute to its reported nutritional and pharmacological properties [76].

### 6.4. Total Flavonoid and Phenolic Content of Mahua

Each extract of total phenolic content was measured using the Folin–Ciocalteu micro-method [31,73]. The results of numerous experiments were represented as milligrams of gallic acid equivalents per gram of fresh weight (mg GAE/g FW), which provided a standardized measure of phenolic content. Each extract was analyzed using the aluminum chloride colorimetric method outlined [58,77]. Total flavonoid content (TFC) was given as milligrams of quercetin equivalents per gram mahua (mg QCE/gFW) [77].

## 7. Potential Market for Mahua-Based Products

Ranjani et al. [52] and Lungade et al. [67] described the utilization of mahua and its uses. Because of its delicacy, tribes utilized the mahua flower to sweeten basic foods, which were part of their daily meals. The sweetness is due to the high reduced sugar content. Flowers are used to sweeten various local cuisines, including halwa, kheer, sweets, puri, and burfi. Ramadan et al. [78] and Lungade et al. [67] developed cooking rice with fresh mahua flowers to produce a nice aroma and perfumes such as 2-acetyl-1-pyrroline (2 AP).

### 7.1. Preparation of Syrup from Mahua Flowers

Different studies have shown that dried mahua flowers can be used to make syrup. This is utilized for fermentation due to its sweetness. The water extraction process is used to prepare syrup. The aqueous extraction results in black syrup. Slaked lime and activated charcoal [3.5–5.0%] were the finest clarifying agents for mahua syrup. Mahua syrup is commonly used as a sweetener in confectionery items and other culinary products, and can be assessed using sensors [53,79].

### 7.2. Liquor Preparation from Mahua Flowers

Lungade et al. [69] prepared the most popular product, distilled liquor from mahua flowers. During the summer months, non-alcoholic beverages made from diverse plant species are commonly used for cooling and refreshment. The mahua flower is utilized in a variety of applications, including distilled liquor, portable spirits, vinegar, and cattle feed [52,53,80].

### 7.3. Jelly, Jam, Marmalade, and Pickle

Different food products have been developed by using guava and mahua, such as jam, jelly, and syrup, in which mahua food products received high ratings for color, flavor, taste, texture, mouthfeel, and overall acceptance. The use of mature, ripe mahua fruit in jam-making with citric acid is reported [35,40]. Guava pulp is used in marmalade, syrup, pickles, and jelly to disguise its astringent flavor, whereas fresh mahua flowers are utilized in the formulation of jam and jelly. The prepared items were examined for color, flavor, and texture for acceptance using the hedonic test. The hedonic test results indicated that all the mahua products were extremely acceptable [53,81]. The process included preparing jelly by first soaking clean mahua flowers in water overnight, then cooking the soaked flowers and crushing them properly with a fruit masher. To prepare the jelly, add pectin and sugar, which need to be mixed evenly to convert it into jelly [32,50,53,82,83]. Nutrient composition alone does not necessarily translate into nutritional impact, as factors such as bioavailability, dietary intake levels, consumer acceptance, and food-processing practices influence actual nutritional outcomes [83].

Despite increasing interest in mahua-based products, commercialization and SWOT analysis remain constrained by irregular supply chains, limited processing infrastructure, inadequate standardization, seasonal availability, regulatory uncertainties, and low consumer awareness beyond traditional consumption regions. Future market expansion will require improved value-chain integration, quality assurance systems, product diversification, branding strategies, and supportive policy frameworks.

## 8. Processing, Biomaterial Engineering, and Value Addition

This section outlines how *Madhuca longifolia* (mahua) biomass can be transformed, using green technologies, into safe and standardized food, nutraceutical, and biomaterial products that address energy deficiency, micronutrient gaps, and obesity-linked non-communicable diseases in an integrated manner.

### 8.1. Turning Mahua into Nutritious Ingredients: Greener Ways to Process and Extract

Recent work shows that mahua flowers and seeds can be processed gently to keep their nutrition intact while limiting chemical inputs. Solar or shade drying at around 40–45 °C for 8–10 h can bring flower moisture down to roughly 8–10%, which is low enough to inhibit mold growth yet preserves most phenolics and sugars. In one open access study, dried flowers contained about 6.37% protein, 0.50% fat, 54.06% total sugars, and 4.46% ash on a dry weight basis, underlining their value as an energy dense, micronutrient bearing ingredient for undernourished groups.

**Figure 3:**
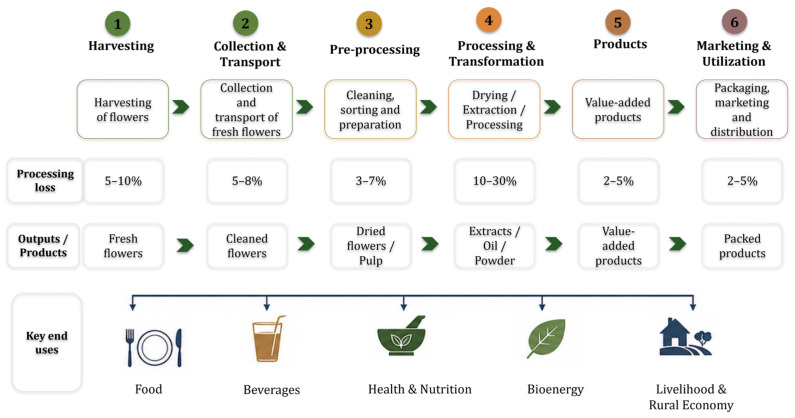
Flowchart illustrating the processing pathways and value-added product development from fresh *Madhuca longifolia* flowers. Figure was prepared using MS-Office powerpoint.

Seed processing also benefits from greener approaches. Cold mechanical pressing followed by supercritical CO_2_ extraction can recover 50–61% oil from mahua kernels while avoiding hexane residues and keeping free fatty acid levels within edible oil standards [80]. Across 37 genotypes, oil iodine values in the range of 52.0–68.6 and saponification values of about 185–198 mg KOH g^−1^ indicate a semi-solid fat suitable for structured foods and nutraceutical delivery systems. Aqueous or hydro ethanolic extractions (40–60% ethanol) of flowers have yielded polyphenol concentrations in the order of tens of mg gallic acid equivalents per gram, offering a realistic basis for antioxidant rich formulations. Figure 3 highlights conventional and emerging utilization approaches, including juice extraction, pulp processing, sun drying, fermentation, confectionery production, beverages, livestock feed, and liquor preparation, demonstrating the economic and nutritional potential of mahua-based products [40].

Table 4 outlines the phytochemical profile of *Madhuca longifolia* and demonstrates the presence of diverse bioactive compounds distributed across different plant parts, which contribute to its broad spectrum of ethnomedicinal and pharmacological properties. Leaves are particularly rich in flavonoids, triterpenoids, sterols, and carotenoids such as quercetin, β-carotene, myricetin, erythrodiol, and oleanolic acid, which are associated with significant antioxidant, antimicrobial, antiulcer, anxiolytic, immunomodulatory, and anticancer activities [84,85,86,87,88,89,90,91,92,93,94,95]. Bark extracts contain several triterpenes and phenolic compounds including ethylcinnamate, α- and β-amyrin acetates, ursolic acid, and α-tocopherol, which exhibit hepatoprotective, anti-inflammatory, antioxidant, immunomodulatory, and antihyperglycemic effects [68,84,86,90,91,93,96,97,98,99,100,101,102]. Seeds are enriched with fatty acids, amino acids, flavonoids, and saponins such as Mi-saponin A and B, contributing to antioxidant, antihyperglycemic, anticonvulsant, and anticancer activities [58,78,85,86,103,104]. Similarly, fruits and flowers contain compounds like quercetin derivatives, β-sitosterol, vitamins A and C, and long-chain alcohols that support antioxidant, antimicrobial, antiulcer, analgesic, and traditional therapeutic applications [105,106,107,108,109,110]. Overall, the presence of these phytoconstituents validates the traditional medicinal importance of *Madhuca longifolia* and highlights its potential as a valuable source of pharmacologically active natural compounds for future therapeutic and biotechnological applications.

**Figure 4:**
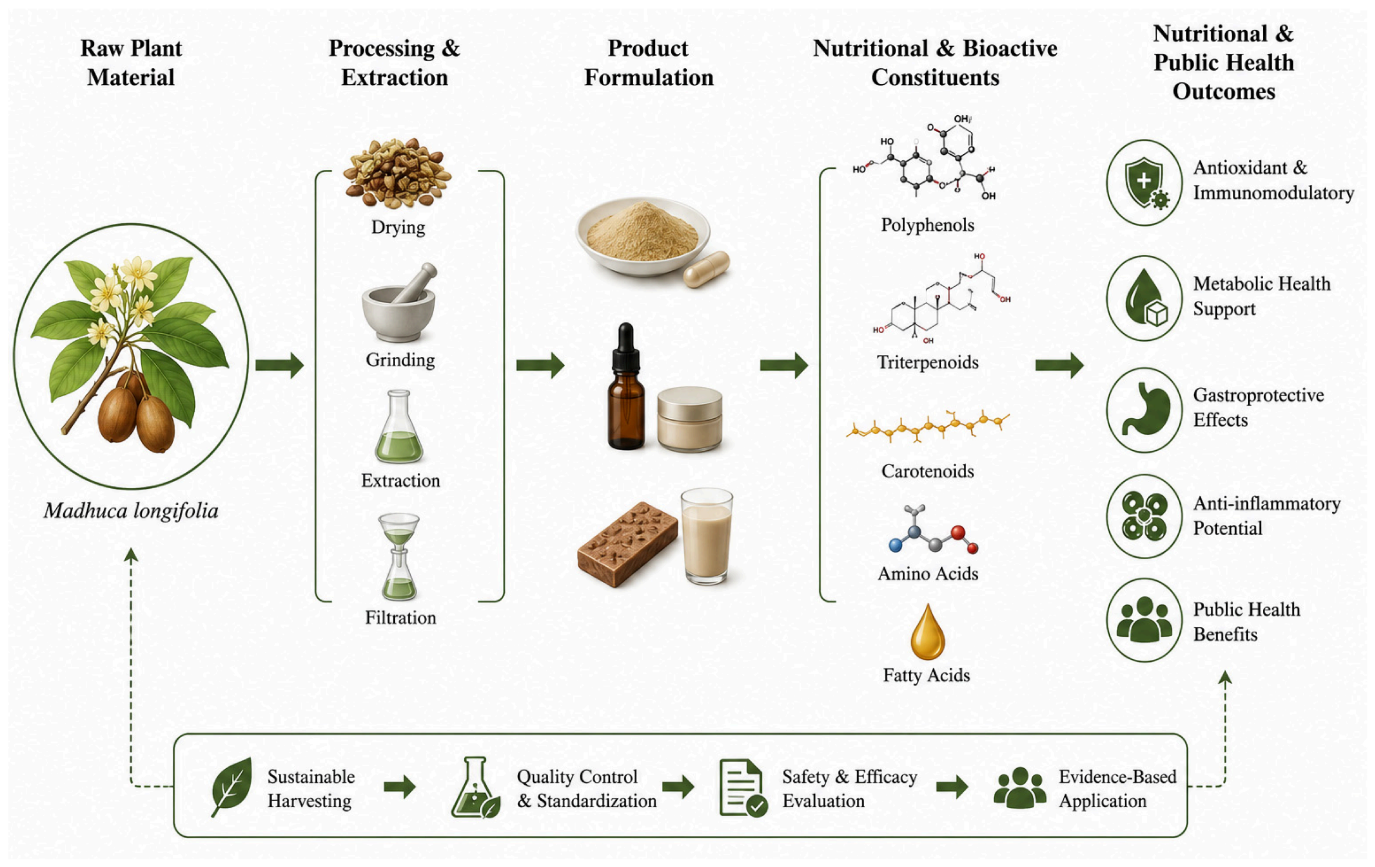
Integrated framework illustrating the valorization pathway of *Madhuca longifolia* from raw plant material to nutritional and public health outcomes. Created in BioRender. Antoniadou Maria. (2026). https://BioRender.com/kqxdz9q (accessed on 25 May 2026).

The framework in Figure 4 depicts the sequential transformation of *Madhuca longifolia* biomass into value-added nutraceutical and functional products. Beginning with raw plant material, harvested plant parts undergo processing steps including drying, grinding, extraction, and filtration to obtain bioactive-rich extracts. These extracts are subsequently incorporated into diverse product formulations such as powders, capsules, liquid extracts, functional foods, and health supplements. The resulting products contain key nutritional and bioactive constituents, including polyphenols, triterpenoids, carotenoids, amino acids, and fatty acids, which contribute to multiple biological functions. These bioactive compounds are associated with antioxidant, immunomodulatory, gastroprotective, anti-inflammatory, and metabolic health-supporting properties, ultimately contributing to broader public health benefits. The lower panel highlights critical enabling factors required for sustainable utilization and commercialization, including sustainable harvesting, quality control and standardization, and efficacy evaluation, and evidence-based application. Collectively, the framework demonstrates the continuum from plant resource utilization to the development of scientifically validated products with potential nutritional, therapeutic, and public health significance.

### 8.2. Designing Mahua-Based Foods and Supplements That People Will Actually Use

Product development studies demonstrate that mahua can be woven into everyday foods without sacrificing taste. When wheat flour in traditional sweets or cookies is partially replaced with 10–30% powdered mahua flowers, the resulting products show higher energy density and micronutrient levels while maintaining overall acceptability scores. One formulation study reported that 15% mahua flower addition raised carbohydrate and mineral content (calcium, iron, and phosphorus) measurably compared with controls, supporting its use in snacks for children and women with undernutrition.

More recent work on “smart” baked products illustrates how mahua extracts can contribute to the double and triple burden of malnutrition. Nutrient-rich muffins prepared with 6% brown top millet flour and 5% mahua flower extract showed significantly higher total phenolics and antioxidant activity than the base formulation, yet remained acceptable to a trained sensory panel over a 60-day shelf-life study. Animal and in vitro models add a functional dimension: mahua seed ethanol extract at oral doses of 10–15 mg kg^−1^ body weight reduced carrageenan-induced paw edema by 16.6–23.7% and saponin fractions at 1.5–3 mg kg^−1^, hence achieving similar anti-inflammatory effects. This suggests that appropriately dosed food or nutraceutical formats could help modulate low grade inflammation linked to obesity and metabolic disease [83].

The biological and health-related effects attributed to different parts of *Madhuca longifolia* are supported by varying levels of scientific evidence and should therefore be interpreted according to the nature of the underlying studies. Traditional medicinal uses of fruits and flowers, including their application for general health promotion, pain relief, ulcer management, and treatment of microbial infections, are primarily based on ethnomedicinal knowledge accumulated through long-term community practices [106,107,108,109,110]. In contrast, many reported antioxidant, antimicrobial, anti-inflammatory, antiulcer, immunomodulatory, and anticancer properties of leaves and bark are largely derived from phytochemical investigations and in vitro bioassays demonstrating the bioactivity of compounds such as quercetin, myricetin, oleanolic acid, β-sitosterol, stigmasterol, α-tocopherol, and amyrin derivatives [68,84,85,86,87,88,89,90,91,92,93,94,95,96,97,98,99,100,101,102]. Similarly, antihyperglycemic, hepatoprotective, anticonvulsant, and immunomodulatory effects associated with bark and seeds have been predominantly validated in experimental animal models, where extracts and isolated phytoconstituents exhibited pharmacological responses under controlled laboratory conditions [58,85,91,96,97,98,99,100,101,102,103,104]. Although these preclinical findings provide important mechanistic insights and support the therapeutic potential of *M. longifolia*, direct clinical evidence in humans remains limited. Therefore, the reported health benefits should be viewed as a continuum of evidence ranging from traditional use and laboratory-based studies to animal experimentation, with a substantial need for well-designed human clinical studies to confirm efficacy, dosage, and long-term therapeutic relevance [58,68,78,84,85,86,87,88,89,90,91,92,93,94,95,96,97,98,99,100,101,102,103,104,105,106,107,108,109,110]. *Madhuca longifolia* aligns well with the principles of sustainable diets and the One Health framework due to its nutritional value, low-input cultivation requirements, and adaptability to marginal environments. As an underutilized indigenous food resource, it can contribute to dietary diversification, food and nutrition security, and the conservation of agro-biodiversity.

### 8.3. Feasibility, Scalability, Commercialization Prospects, and Regulatory Considerations

*Madhuca longifolia* possesses considerable potential as a functional food and nutraceutical resource due to its wide availability, high flower yield, nutritional richness, and long history of traditional consumption. The development of value-added products such as beverages, confectioneries, bakery products, and dietary supplements demonstrates its commercial feasibility and potential to support rural livelihoods and tribal economies [78]. Furthermore, the plant’s adaptability to marginal environments and low-input cultivation systems favor large-scale production and sustainable utilization. However, successful commercialization requires addressing challenges related to variability in phytochemical composition, post-harvest handling, quality standardization, and product shelf-life. The establishment of standardized processing protocols and quality control measures is essential to ensure product consistency and consumer acceptance [48]. Regulatory approval is another critical requirement, particularly for products marketed with health claims. Compliance with food safety regulations, nutraceutical guidelines, and novel food frameworks established by agencies such as FSSAI and international regulatory bodies will be necessary for broader market access [111,112]. Future efforts should focus on clinical validation, and evidence-based product development to facilitate the transition of *M. longifolia* from a traditionally utilized forest resource to a globally recognized functional food ingredient.

Toxicological studies provide additional reassurance. In rodent models, seed and flower extracts, administered orally at doses several fold higher than probable human intakes, did not produce significant changes in body weight gain, organ weights, or liver enzyme activities, and histology remained within normal limits, suggesting a wide margin of safety when human serving sizes are kept in the tens to a few hundreds of milligrams of extract per day [113]. Aligning products with the Food Safety and Standards Authority of India (FSSAI), such as capping added sugars to stay within “low sugar” claims and declaring exact amounts of energy, protein, iron, zinc, and key phytochemicals per serving will be essential if mahua-based foods and supplements are to be mainstreamed into school feeding, ICDS, and adult NCD prevention programs targeting the triple burden of malnutrition [114]. While mahua possesses considerable nutritional and industrial potential, comprehensive safety evaluation remains essential for wider utilization. Existing studies indicate the need for further assessment of toxicological parameters, anti-nutritional constituents, potential allergenicity, and long-term consumption effects. In addition, regulatory requirements for food, nutraceutical, pharmaceutical, and biomaterial applications vary across jurisdictions and may influence commercial adoption. Future research should prioritize standardized safety assessments and regulatory compliance to facilitate sustainable product development.

## 9. Conclusions

This review highlights *Madhuca longifolia* as a nutritionally valuable indigenous plant with documented traditional food uses and a diverse profile of bioactive compounds. Available studies indicate that mahua flowers, seeds, fruits, and other plant parts contain carbohydrates, fatty acids, amino acids, vitamins, polyphenols, and triterpenoids that may contribute to their nutritional and functional properties. However, evidence supporting many of the reported health benefits is derived primarily from in vitro and animal studies, while human clinical data remain limited.

*Madhuca longifolia* possesses promising nutritional, functional, ethnomedicinal, and industrial attributes that may contribute to strategies addressing aspects of the triple burden of malnutrition. Its edible flowers provide dietary energy and valuable phytochemicals, while various plant parts offer opportunities for functional food development and value-added applications. Therefore, mahua should be viewed as a potentially valuable component of diversified and sustainable food systems rather than a standalone solution to malnutrition.

## Figures and Tables

**Table 1:** Composition of amino acids in mahua flowers [30].

Amino Acids	g/100 g
Threonine	5.86
Tyrosine	3.94
Valine	7.25
Cystine	3.35
Isoleucine	7.91
Methionine	1.8
Leucine	12.98
Phenylalanine	6.4
Lysine	4.67

**Table 2:** Functional, medicinal, and nutraceutical relevance of *Madhuca longifolia* plant parts with supporting traditional and experimental evidence.

Plant Part	Medicinal Properties	Representative Uses	References
Flowers	Anti-inflammatory; antioxidant; antidiabetic; antiulcer; antidiarrhoeal; cardioprotective; galactagogue; hepatoprotective	Ayurveda uses for cooling, digestion, lactation; extracts show antioxidant, glucose lowering, and lipid improving effects	[37,60,61]
Seeds/seed oil	Analgesic; anti-arthritic; anti-inflammatory; emollient; laxative; wound healing	Topical use for skin disorders and rheumatism; oral mild laxative; experimental anti-inflammatory and anti-arthritic activity	[60,62]
Bark	Astringent; antidiarrhoeal; antihaemorrhoidal; antiulcer; antimicrobial	Decoctions for diarrhea, piles, gum and throat infections; antibacterial and antiulcer activity reported	[37,57]
Leaves	Antimicrobial; anti helminthic; anti-inflammatory; wound healing; antidiabetic	Poultices for wounds and skin diseases; juice for worms and itching; hypoglycemic effects in studies	[37,61]
Whole tree	Antioxidant; antidiabetic; cardioprotective; hepatoprotective; nutraceutical	Source of phenolics, flavonoids, saponins and minerals for functional foods and nutraceuticals	[60,61]

**Table 3:** Nutritional value of mahua flower [64].

Sr.no	Constituents	Mahua Flower
1	Moisture	11.61–19.8
2	pH	4.6
3	Fat (%)	0.06–0.09
4	Protein (%)	5.62
5	Total sugars (g/100 g)	41.62

**Table 4:** Major phytoconstituents identified from different parts of *Madhuca longifolia* and their reported ethnomedicinal and pharmacological activities.

Plant Part/ Part(s) Used	Major Phytoconstituents/Bioactive Compounds	Ethnomedicinal/Pharmacological Activity	Reference(s)
Leaves	Quercetin, β-carotene, erythrodiol, palmitic acid, myricetin, xanthophylls, oleanolic acid, β-sitosterol, stigmasterol, n-octacosanol	Antioxidant activity	[84,85,86,87,88]
Leaves	β-carotene, xanthophylls, erythrodiol, palmitic acid, myricetin derivatives, quercetin-3-galactoside, oleanolic acid, β-sitosterol derivatives, stigmasterol, n-hecacosanol, n-octacosanol	Antioxidant, antimicrobial, antiulcer, anxiolytic, immunomodulatory, anticancer activities	[89,90,91,92,93,94,95]
Bark	Ethylcinnamate, sesquiterpene alcohol, α-amyrin acetates, β-amyrin acetates, α-tocopherol	Antioxidant, hepatoprotective, antimicrobial, anti-inflammatory, antihyperglycemic activities	[86,93,96,97,98,99]
Bark	21-Hydroxy-3-oleanyl myricitate, ursolic acid, n-hexyl-3-acetyl betulinate, 3-(27-carboxy oleanyl)-octanate	Immunomodulatory and hepatoprotective activities	[91,96]
Bark	Ethylcinnamate, sesquiterpene alcohol, α-tocopherol, 3β-monocaprylic ester of erythrodiol, 3β-capryloxy oleanolic acid, α- and β-amyrin acetates	Anti-inflammatory and antioxidant activities	[68,84,90,93,100,101,102]
Seeds	Arachidic acid, stearic acid, aspartic acid, isoleucine, leucine, cysteine, α-alanine, proline, threonine	Antioxidant and antihyperglycemic activities	[64,103,104]
Seeds	Arachidic, linoleic, oleic, myristic, palmitic, stearic acids, amino acids, myricetin, quercetin, Mi-saponin A and B	Anticancer and anticonvulsant activities	[58,78,85,86,103]
Fruits	3β-D-glucoside, n-hexacosanol	Anticancer activity	[105]
Fruits	n-hexacosanol, quercetin, dihydroquercetin, β-sitosterol derivatives, α- and β-amyrin acetates	Traditional medicinal and antioxidant uses	[106,107,108]
Flowers	Vitamin A, vitamin C	Analgesic, antiulcer, antimicrobial activities	[109,110]

## Data Availability

No new data were created or analyzed in this study. Data sharing is not applicable to this article.

## References

[1] Kiosia A., Dagbasi A., Berkley J.A., Wilding J.P.H., Prendergast A.J., Li J.V., Swann J., Mathers J.C., Kerac M., Morrison D. (2024). The double burden of malnutrition in individuals: Identifying key challenges and re-thinking research focus. Nutr. Bull..

[2] Hegazi R., Miller A., Sauer A. (2024). Evolution of the diagnosis of malnutrition in adults: A primer for clinicians. Front. Nutr..

[3] Mark H.E., da Costa G.D., Pagliari C., Unger S.A. (2020). Malnutrition: The silent pandemic. BMJ.

[4] Sagastume D., Barrenechea-Pulache A., Ruiz-Alejos A., Polman K., Beňová L., Ramírez-Zea M., Peñalvo J.L. (2024). Quantifying overlapping forms of malnutrition across Latin America: A systematic literature review and meta-analysis of prevalence estimates. Adv. Nutr..

[5] Keeley B., Little C., Zuehlke E. (2019). The State of the World’s Children 2019: Children, Food and Nutrition—Growing Well in a Changing World.

[6] Von Haehling S., Anker M.S., Anker S.D. (2016). Prevalence and clinical impact of cachexia in chronic illness in Europe, USA, and Japan: Facts and numbers update 2016. J. Cachexia Sarcopenia Muscle.

[7] Ethgen O., Beaudart C., Buckinx F., Bruyere O., Reginster J.Y. (2017). The future prevalence of sarcopenia in Europe: A claim for public health action. Calcif. Tissue Int..

[8] Lusting R.H., Collier D., Kassotis C., Roepke T.A., Kim M.J., Blanc E., Barouki R., Bansal A., Cave M.C., Chatterjee S. (2020). Obesity I: Overview and molecular and biochemical mechanisms. Biochem. Pharmacol..

[9] Varghese J.S., Stein A.D. (2019). Malnutrition among women and children in India: Limited evidence of clustering of underweight, anemia, overweight, and stunting within individuals and households at both state and district levels. Am. J. Clin. Nutr..

[10] Wells J.C., Sawaya A.L., Wibaek R., Mwangome M., Poullas M.S., Yajnik C.S., Demaio A. (2020). The double burden of malnutrition: Etiological pathways and consequences for health. Lancet.

[11] Victora C.G., Christian P., Vidaletti L.P., Gatica-Domínguez G., Menon P., Black R.E. (2021). Revisiting maternal and child undernutrition in low-income and middle-income countries: Variable progress towards an unfinished agenda. Lancet.

[12] Ji N., Kumar A., Joe W., Kuriyan R., Sethi V., Finkelstein J.L., Mehta S. (2024). Prevalence and correlates of double and triple burden of malnutrition among children and adolescents in India: Comprehensive National Nutrition Survey. J. Nutr..

[13] Zhang X., Liu J., Ni Y., Yi C., Fang Y., Ning Q., Shen B., Zhang K., Liu Y., Yang L. (2024). Global prevalence of overweight and obesity in children and adolescents: A systematic review and meta-analysis. JAMA Pediatr..

[14] Black M.M. (1998). Zinc deficiency and child development. Am. J. Clin. Nutr..

[15] Luhar S., Mallinson P.A.C., Clarke L., Kinra S. (2018). Trends in the socioeconomic patterning of overweight/obesity in India: A repeated cross-sectional study using nationally representative data. BMJ Open.

[16] Patel R., Srivastava S., Kumar P., Chauhan S. (2020). Factors associated with double burden of malnutrition among mother-child pairs in India: A study based on National Family Health Survey 2015–16. Child. Youth Serv. Rev..

[17] Kumar P., Chauhan S., Patel R., Srivastava S., Bansod D.W. (2021). Prevalence and factors associated with triple burden of malnutrition among mother-child pairs in India: A study based on National Family Health Survey 2015–16. BMC Public Health.

[18] Varghese J.S., Gupta A., Mehta R.D., Stein A.D., Patel S.A. (2022). Changes in child undernutrition and overweight in India from 2006 to 2021: An ecological analysis of 36 states. Glob. Health Sci. Pract..

[19] Nichol L. (2003). Local food production: Some implications for planning. Plan. Theory Pract..

[20] Panghal A., Sindhu S., Sarao J., Dahiya S. (2024). Local food entrepreneurship in rural India: Modelling the challenges. Int. J. Rural Manag..

[21] Mars M.M., Schau H.J. (2017). Institutional entrepreneurship and the negotiation and blending of multiple logics in the Southern Arizona local food system. Agric. Hum. Values.

[22] Tkemaladze J. (2025). Concept to the food security. Longev. Horiz..

[23] World Obesity Federation (2024). World Obesity Atlas 2023.

[24] Monteiro C.A., Cannon G., Moubarac J.C., Levy R.B., Louzada M.L.C., Jaime P.C. (2018). The UN Decade of Nutrition, the NOVA food classification and the trouble with ultra-processing. Public Health Nutr..

[25] Devarakonda S. (2026). On Mahua, its people and the markets: Reflections from Bastar, Chhattisgarh. For. Trees Livelihoods.

[26] Pinakin D.J., Kumar V., Kumar A., Gat Y., Suri S., Sharma K. (2018). Mahua: A boon for pharmacy and food industry. Curr. Res. Nutr. Food Sci. J..

[27] Akash S.I., Yasmin S., Jahan I.T., Aziz M.G. (2025). Production scales and industrial production processes of dietary supplements and nutraceuticals: An overview. Dietary Supplements and Nutraceuticals.

[28] Jha D., Mazumder P.M. (2018). Biological, chemical and pharmacological aspects of *Madhuca longifolia*. Asian Pac. J. Trop. Med..

[29] Purwar S., Bisht V., Gupta E. (2025). MAHUA (*Madhuca indica*): The multifunctional tree of tradition and sustenance. Fasting Superfoods: Cultivation, Nutrition & Market Potential.

[30] Khutade K., Shah H., Joshi V., Chanda S. (2022). Traditional brewing techniques for production of bioethanol from *Madhuca longifolia* flowers using isolated strain VIMS 1, *Saccharomyces cerevisiae* ATCC-9763 and study of parameters. Int. J. Adv. Res. Biol. Sci..

[31] Kumari P., Bhargava B. (2021). Phytochemicals from edible flowers: Opening a new arena for healthy lifestyle. J. Funct. Foods.

[32] Jamir R. (2024). More than a country liquor: *Madhuca longifolia* as a facilitator of SDGs. Transformative Practices in Archaeology: Empowering Communities and Shaping Sustainable Futures.

[33] Saha D., Sarankar S.K. (2023). Critical review on potentials of ethnopharmacological, ethnomedicinal and traditional practices of *Madhuca longifolia* (J. Koenig Ex L.) J.F. Macbr. (Family: Sapotaceae). Prospect. Pharm. Sci..

[34] Sangameswaran B., Saluja M.S., Sharma A. (2012). Anticancer activity of ethanol extract of *Madhuca longifolia* against Ehrlich Ascites Carcinoma. Mol. Clin. Pharmacol..

[35] Santhoshini C.N.R., Jagadeeswari V.V., Makwana R.J., Pradhan R., Burdak M.K., Mahesh P., Nagaraju V., Krushnaji R.T. (2025). A comprehensive review on edible flowers: The sustainable solution for food security and nutrition. Eur. J. Nutr. Food Saf..

[36] Patel M., Pradhan R., Naik S. (2011). Physical properties of fresh mahua. Int. Agrophysics.

[37] Dwivedi A., Priyadarshini A., Induar S. (2022). Mahua (*Madhuca longifolia*) flower and its application in food industry: A review. Int. J. Chem. Stud..

[38] Boyce P.C. (2009). A review of *Pothos* L. (Araceae: Pothoideae: Pothoeae) for Thailand. Thai For. Bull. Bot..

[39] Oyebode O., Kandala N.B., Chilton P.J., Lilford R.J. (2016). Use of traditional medicine in middle-income countries: A WHO-SAGE study. Health Policy Plan..

[40] Dubey I., Chhajed M., Chourasiya R., Chouhan P.S., Walvekar A., Bhandari Y., Verma K. (2023). A review of the botanical, conventional applications, phytochemical constituents, and pharmacology of *Madhuca longifolia* (Koenig) J.F. Macbr. Pharmacogn. Rev..

[41] Samkaria S., Kumari P. (2025). Wild edible flowers of Indian Himalayan region, their traditional uses and potential health benefits: A way forward for food and nutritional security. Plant Foods Hum. Nutr..

[42] Ramadan M.F., Abdel-Hamed E.M.W. (2020). Health-promoting potential and nutritional value of *Madhuca longifolia* seeds. Nuts and Seeds in Health and Disease Prevention.

[43] Singh V., Singh J., Kushwaha R., Singh M., Kumar S., Rai A.K. (2020). Assessment of antioxidant activity, minerals and chemical constituents of edible mahua. Nutr. Food Sci..

[44] Jha S., Vaibhav V., Suneetha V. (2013). A culinary mahua (*Madhuca indica*) flower from Bihar, India—A potential in production of jam, alcohol for pharmacological benefits with fertilizer value. Int. J. Drug Dev. Res..

[45] Jurri R., Kumar A., Mollick F. (2022). Medicinal importance of mahua among Gond, Halba and Dorla tribes of Bijapur district of Chhattisgarh. J. Bio Innov..

[46] Khare P., Kishore K., Sharma D.K. (2018). Medicinal uses, phytochemistry and pharmacological profile of *Madhuca longifolia*. Asian J. Pharm. Clin. Res..

[47] Saxena V., Jasrotia A., Sharma S., Khapre M. (2025). Double burden of malnutrition as a rising public health problem: Indian scenario. Handbook of Public Health Nutrition: International, National, and Regional Perspectives.

[48] Rao K.M., Kumar R.H., Krishna K.S., Bhaskar V., Laxmaiah A. (2015). Diet & nutrition profile of Chenchu population-a vulnerable tribe in Telangana & Andhra Pradesh, India. Indian J. Med. Res..

[49] Nayak S., Sahoo U.K. (2021). Role of non-timber forest products from *Madhuca latifolia* in enhancing local livelihoods and household dependency in Odisha. Diversity and Dynamics in Forest Ecosystems.

[50] Sreenivasa Rao J., Patil P.B., Goudar G., Jaleel A., Qadri S.S.Y.H. (2025). Mahua (*Madhuca indica*): Indigenous flower as nutrient and phenolic rich food to combat anaemia and associated disorders. Phytochem. Rev..

[51] Singh V. (2017). Phenolic content and antioxidant activity of solvent extracts of mahua (*Madhuca longifolia*) flowers and fruit. Int. J. Nutraceuticals Funct. Foods Nov. Foods.

[52] Mishra A., Poonia A. (2019). Mahua (*Madhuca longifolia*) flowers: Review on processing and biological properties. Nutr. Food Sci..

[53] Bisht V., Solanki V.K., Dalal N. (2018). Mahua an important Indian species: A review. J. Pharmacogn. Phytochem..

[54] Jarapala S.R., Shivudu G., Mangathya K., Rathod A., Panda H., Reddy P.K. (2021). A new approach for identifying potentially effective indigenous plants consumed by Chenchu tribes and their nutritional composition—India. Am. J. Plant Sci..

[55] Gahukar R.T. (2018). Plant-based traditional food for nutritional security in India: Need, issues and challenges. Everyman’s Sci..

[56] Bhatia H., Sharma Y.P., Manhas R.K., Kumar K. (2018). Traditionally used wild edible plants of district Udhampur, J&K, India. J. Ethnobiol. Ethnomed..

[57] Kaur N., Singh N., Garg M. (2025). Mahua’s adaptogenic nature: Benefits and mechanisms. Phytoadaptogenic Properties of Medicinal Plants.

[58] Sunita M., Sarojini P. (2013). *Madhuca lonigfolia* (Sapotaceae): A review of its traditional uses and nutritional properties. Int. J. Humanit. Soc. Sci. Invent..

[59] Patel P.K., Janghel V., Chandel S.S., Sahu J. (2019). *Madhuca indica* (Mahua)—Pharmaceutical, nutraceutical and economical importance for tribal people of Chhattisgarh state. Int. J. Pharm. Phytopharm. Res..

[60] Sameeksha S., Puneet G., Ragini G. (2025). Exploring the bioactive-based therapeutic and culinary potential of *Madhuca longifolia* flower and delineating the research gaps in terms of scientific validation. J. Food Sci. Technol..

[61] Pant S., Samant (2006). Diversity, distribution, uses and conservation status of plant species of the Mornaula Reserve Forests, West Himalaya, India. Int. J. Biodivers. Sci. Manag..

[62] Viguiliouk E., Glenn A.J., Nishi S.K., Chiavaroli L., Seider M., Khan T., Bonaccio M., Iacoviello L., Mejia S.B., A Jenkins D.J. (2019). Associations between dietary pulses alone or with other legumes and cardiometabolic disease outcomes: An umbrella review and updated systematic review and meta-analysis of prospective cohort studies. Adv. Nutr..

[63] Adhikari S., Adhikari J. (1989). Sal olein and Mahua olein for direct edible use. J. Am. Oil Chem. Soc..

[64] Chacko S.I., Narvekar V.N. (2023). Unlocking the power of Mahua: A comprehensive guide. IOSR J. Biotechnol. Biochem..

[65] Bano S., Awasthi M., Tripathi A.D., Agarwal A., Nandan A. (2025). Formulation and shelf life evaluation of nutrient rich *Urochloa ramosa* muffins enriched with *Madhuca longifolia* extract. Sci. Rep..

[66] Mohan K.R. (2025). Tribal knowledge in sustainable food systems: Investigating agriculture, nutrition, and culinary traditions. Tribal Knowledge Systems.

[67] Lungade P., Karadbhajne S.V. (2022). Mahua flower (*Madhuca indica*): Approach of functional, nutritional characteristics and an accompaniment to food products. J. Pharm. Negat. Results.

[68] Kureel R.S., Kishor R., Dutt D., Pandey A. (2009). Mahua: A Potential Tree Borne Oilseed.

[69] Lungade P., Karadbhajne S. (2024). Sustainable utilization of Mahua flowers: A multi-product approach for tribal economic upliftment. Indian J. Agric. Biochem..

[70] Cummings J.H., Stephen A.M. (2007). Carbohydrate terminology and classification. Eur. J. Clin. Nutr..

[71] World Health Organization (2023). Carbohydrate Intake for Adults and Children: WHO Guideline.

[72] Sarkar N., Chatterjee B.P. (1983). Some structural features of the polysaccharide of mahua (*Madhuca indica*) flowers. Carbohydr. Res..

[73] Cicco N., Lanorte M.T., Paraggio M., Viggiano M., Lattanzio V. (2009). A reproducible, rapid and inexpensive Folin–Ciocalteu micro-method in determining phenolics of plant methanol extracts. Microchem. J..

[74] Mishra M., Pattnaik S., Singh H., Naik P.K. (2023). Exploring the role of Mahua as a functional food and its future perspectives. Recent Frontiers of Phytochemicals.

[75] Patel P.K., Prajapati N.K., Dubey B.K. (2012). *Madhuca indica*: A review of its medicinal property. Int. J. Pharm. Sci. Res..

[76] Jebaseelan S., Ramasubramanian P. (2014). Screening of Madhuca indica for Antidiabetic Activity in Alloxan Induced Diabetic Rats. Res. J. Pharm. Technol..

[77] Seruji N.M.U., Mian V.J.Y., Zamakshari N.H., Karunakaran T., Koo L.F., Jin W.T., Huat C.T. (2023). Antioxidant potential of *Calophyllum gracilentum*: A study on total phenolic content, total flavonoid content, and free radical scavenging activities. J. Angiother..

[78] Ramadan M.F., Mohdaly A.A.A., Assiri A.M., Tadros M., Niemeyer B. (2016). Functional characteristics, nutritional value and industrial applications of *Madhuca longifolia* seeds: An overview. J. Food Sci. Technol..

[79] Sandeep K., Vinti S., Awadhesh K.R. (2025). Rating of various mahua (*Madhuca longifolia*) cookies and their characteristics by sensory evaluation using fuzzy logic. Int. J. Nutraceuticals Funct. Foods Nov. Foods.

[80] Yadav S., Suneja P., Hussain Z., Abraham Z., Mishra S.K. (2011). Genetic variability and divergence studies in seed and oil parameters of mahua (*Madhuca longifolia* Koenig) J.F. Macribide accessions. Biomass Bioenergy.

[81] Thilakarathna R.C.N., Lee F.S., Siow T.F., Tang T.-K., Cheong L.-Z., Lee Y.Y. (2025). Mahua oil fraction: A sustainable and functional 1,3-dipalmitoyl-2-oleoylglycerol (POS)-enriched cocoa butter equivalent for chocolate production. Food Chem..

[82] Passi S.J. (2024). Dietary guidelines for Indians-2024: A critical appraisal. J. Epidemiol. Found. India.

[83] Hotz C., Gibson R.S. (2007). Traditional food-processing and preparation practices to enhance the bioavailability of micronutrients in plant-based Diets1. J. Nutr..

[84] Chakma C.S. (2011). Pharmacological screening of isolated compound from seeds of *Madhuca longifolia* gives significant analgesic effect. Int. Res. J. Pharm..

[85] Inganakal T.S., Swamy P.L. (2013). Evaluation of in vitro antioxidant activity of a triterpene isolated from *Madhuca longifolia* leaves. Int. J. Pharm. Pharm. Sci..

[86] Agrawal S., Kulkarni G.T., Sharma V.N. (2011). A comparative study on the antioxidant activity of methanolic extracts of *Terminalia paniculata* and *Madhuca longifolia*. Free. Radic. Antioxid..

[87] Kumar A., Biswas K., Setty S.R. (2013). Evaluation of the antioxidant and hepatoprotective activity of *Madhuca longifolia* (Koenig) leaves. Indian J. Res. Pharm. Biotechnol..

[88] Palani S., Raja S., Karthi S., Archana S., Kumar B.S. (2010). In vivo analysis of nephro- and hepatoprotective effects and antioxidant activity of *Madhuca longifolia* against acetaminophen-induced toxicity and oxidative stress. J. Pharm. Res..

[89] Khare C.P. (2000). Rational western therapy, ayurvedic and other traditional usage. Encyclopedia of Indian Medicinal Plants.

[90] Rajagopal P.L., Dhilna K.K., Kumar S.P.N., John J. (2013). Herbs in inflammation: A review. Int. J. Ayurvedic Herb. Med..

[91] Alexander J., Atli G., Bentord D. (2009). Saponin in *Madhuca longifolia* as undesirable substance in animal feed. Eur. Food Saf. Auth..

[92] Liang Y.Z., Xie P., Chan K. (2004). Quality control of herbal medicines. J. Chromatogr. B.

[93] Yadav P., Singh D., Mallik A., Nayak S. (2012). *Madhuca longifolia* (Sapotaceae): A review of its traditional uses, phytochemistry and pharmacology. Int. J. Biol. Med. Res..

[94] Drury H. (1986). The Useful Plants of India.

[95] Bulbul I.J., Begum Y. (2014). Antibacterial, cytotoxic and antioxidant activities of *Madhuca indica*. Sci. Res. J..

[96] Roy S.P., Shirode D., Patel T., Shastry C.S., Gheewala N., Sonara G., Setty S.R., Rajendra S.V. (2010). Antioxidant and hepatoprotective activity of *Madhuca longifolia* (Koenig) bark against CCl4-induced hepatic injury in rats: In vitro and in vivo studies. Res. J. Pharm. Biol. Chem. Sci..

[97] Prashanth S., Kumar A.A., Madhu B., Kumar Y.P. (2010). Antihyperglycemic and antioxidant activity of ethanolic extract of *Madhuca longifolia* bark. Int. J. Pharm. Sci. Rev. Res..

[98] Dahake A.P., Chakma C., Joshi D., Chakma R., Tripathi A. (2010). Antioxidant activity of methanolic extract of *Madhuca longifolia* bark. J. Pharm. Res..

[99] Shekhawat N., Vijayvergia R. (2010). Investigation of anti-inflammatory, analgesic and antipyretic properties of *Madhuca indica* Gmel. Int. J. Mol. Med. Adv. Sci..

[100] Akshatha K.N., Murthy S.M., Lakshmidevi N. (2013). Ethnomedical uses of *Madhuca longifolia*: A review. Int. J. Life Sci. Pharm. Res..

[101] Yoshikawa K., Tanaka M., Arihara S., Pal B.C., Roy S.K., Matsumura E., Katayama S. (2000). New oleanene triterpenoid saponins from *Madhuca longifolia*. J. Nat. Prod..

[102] Shirode D., Patel T., Roy S.P., Setty R.S., Rajendra S.V. (2008). Anti-inflammatory activity of 70% ethanolic extract of *Albizzia lebbeck* leaves and *Madhuca longifolia* bark. Int. J. Pharm. Biol. Sci..

[103] Sengupta A., Choudhury R.S.K. (1978). Triglyceride composition of *Madhuca butyraceae* seed fat. J. Am. Oil Chem. Soc..

[104] Umadevi U., Kamalam M. (2015). Screening of an indigenous medicinal plant, *Madhuca longifolia*, for its antioxidant and antimicrobial properties. Int. J. Pharm. Sci. Res..

[105] Vohra A., Kaur H. (2011). Chemical investigation of medicinal plant *Ajuga bracteosa*. J. Nat. Prod. Plant Resour..

[106] Saikia B. (2006). Ethnomedicinal plants from Gohpur of Sonitpur District, Assam. Indian J. Tradit. Knowl..

[107] Panghal M., Arya V., Yadav S., Kumar S., Yadav J.P. (2010). Indigenous knowledge of medicinal plants used by Saperas community of Khetawas, Jhajjar District, Haryana, India. J. Ethnobiol. Ethnomed..

[108] Dubey N.K., Kumar R., Tripathi P. (2004). Global promotion of herbal medicine: India’s opportunity. Curr. Sci..

[109] Hoffman F.A., Leaders F.E. (1996). Botanical (herbal) medicine in health care: A review from a regulatory perspective. Pharm. News.

[110] Chandra D. (2001). Analgesic effect of aqueous and alcoholic extracts of *Madhuca longifolia* (Koenig). Indian J. Pharmacol..

[111] Jodh R., Tawar M., Kachewar A., Mahanur V., Sureka Y., Atole V. (2022). Pharmacological review on *Madhuca longifolia*. Asian J. Res. Pharm. Sci..

[112] Ahirwar S.D., Singh N., Sagar P. (2023). Development and sensory evaluation of value added product from Mahua (Madhuca longifolia) flower. Int. J. Adv. Res. Innov. Ideas Educ..

[113] Rukmini C. (1990). Reproductive toxicology and nutritional studies on mahua oil (*Madhuca latifolia*). Food Chem. Toxicol..

[114] Pande B., Chandorkar S., Singh M. (2025). A decadal transition in food labelling compliance with the Food Safety and Standards Authority of India regulations and the nutritional composition of processed packaged foods: Implications for public health. World Nutr..

